# Genetic Response of Rat Supraspinatus Tendon and Muscle to Exercise

**DOI:** 10.1371/journal.pone.0139880

**Published:** 2015-10-08

**Authors:** Sarah Ilkhanipour Rooney, John W. Tobias, Pankti R. Bhatt, Andrew F. Kuntz, Louis J. Soslowsky

**Affiliations:** 1 McKay Orthopaedic Research Laboratory, University of Pennsylvania, Philadelphia, Pennsylvania, United States of America; 2 Molecular Profiling Core, Perelman School of Medicine, University of Pennsylvania, Philadelphia, Pennsylvania, United States of America; Queen Mary University of London, UNITED KINGDOM

## Abstract

Inflammation is a complex, biologic event that aims to protect and repair tissue. Previous studies suggest that inflammation is critical to induce a healing response following acute injury; however, whether similar inflammatory responses occur as a result of beneficial, non-injurious loading is unknown. The objective of this study was to screen for alterations in a subset of inflammatory and extracellular matrix genes to identify the responses of rat supraspinatus tendon and muscle to a known, non-injurious loading condition. We sought to define how a subset of genes representative of specific inflammation and matrix turnover pathways is altered in supraspinatus tendon and muscle 1) acutely following a single loading bout and 2) chronically following repeated loading bouts. In this study, Sprague-Dawley rats in the acute group ran a single bout of non-injurious exercise on a flat treadmill (10 m/min, 1 hour) and were sacrificed 12 or 24 hours after. Rats in the chronic group ran 5 days/wk for 1 or 8 weeks. A control group maintained normal cage activity. Supraspinatus muscle and tendon were harvested for RNA extractions, and a custom Panomics QuantiGene 2.0 multiplex assay was used to detect 48 target and 3 housekeeping genes. Muscle/tendon and acute/chronic groups had distinct gene expression. Components of the arachidonic acid cascade and matrix metalloproteinases and their inhibitors were altered with acute and chronic exercise. Collagen expression increased. Using a previously validated model of non-injurious exercise, we have shown that supraspinatus tendon and muscle respond to acute and chronic exercise by regulating inflammatory- and matrix turnover-related genes, suggesting that these pathways are involved in the beneficial adaptations to exercise.

## Introduction

During exercise, muscle and tendon adapt to benefit from the training. This adaptation may present as protein or organizational changes that improve the mechanics of the tissue in the desired loading condition. Acute inflammation is a complex biologic event that aims to protect and repair tissue by initiating protein changes. Two important processes related to inflammation are activation of the arachidonic acid (AA) cascade and degradation of matrix proteins by matrix metalloproteinases (MMPs). In the AA cascade, AA is converted by cyclooxygenase (COX) to prostaglandins, prostacyclins, or thromboxane or by 5-lipoxygenase to leukotrienes. Prostaglandins can mediate blood flow to the tissue [1] and upregulate MMP expression [2]. MMPs and their inhibitors, tissue inhibitors of metalloproteinases (TIMPs), are responsible for matrix turnover and if not carefully balanced can result in excess fibrosis or degeneration [3]. Also intricately weaved into the inflammatory response are cytokines that can regulate and be regulated by AA cascade components and MMPs. A combination of these cytokines, components of the AA cascade, and MMPs may cause the downward spiral that initiates muscle and tendon degeneration; however, they may also be required for beneficial adaptations to exercise. Whether inflammation is a physiologic response to load or pathologic in early tendon and muscle degeneration is unknown.

Previous studies suggest that inflammation plays an important role in the regeneration of muscle and tendon following acute injury [4]; however, whether similar inflammatory responses occur as a result of beneficial, non-injurious loading is unknown. If the appropriate balance in acute inflammation is not achieved, the tissue may not be able to adapt, resulting in injury. Identifying the response of healthy tissue to known, non-injurious loading conditions would help distinguish detrimental and beneficial inflammation.

The overall objective of this study was to screen for alterations in a subset of inflammatory and extracellular matrix genes to identify the responses of rat supraspinatus tendon and muscle to a physiologically relevant, non-injurious loading condition. Specifically, we sought to define how a subset of genes representative of specific inflammation and matrix turnover pathways is altered in supraspinatus tendon and muscle 1) acutely following a single bout of loading and 2) chronically following repeated loading bouts. Our global hypothesis was that a mild inflammatory response is a normal, physiologic requirement for muscle and tendon to adapt to load. Specifically, 1) a mild inflammatory response (changes in AA cascade) in the tendon and muscle would quickly resolve by 24 hours after a single bout of loading, and 2) the tissue will show adaptive matrix changes such as increased collagen production and MMP/TIMP changes indicating matrix turnover with chronic loading.

## Materials and Methods

### Ethics Statement

This study was approved by the University of Pennsylvania’s Institutional Animal Care and Use Committee (protocol 805151). The rats were housed in an AALAC accredited facility that maintained a 12/12 hour light/dark cycle, temperatures between 20–26°C, and humidity between 30–70%, as described in the Guide for Care and Use of Laboratory Animals [5]. Animals were carefully monitored during each exercise session. All animals were euthanized with controlled flow-rate carbon dioxide.

### Study Design and Treadmill Protocol

**Fig 1:**
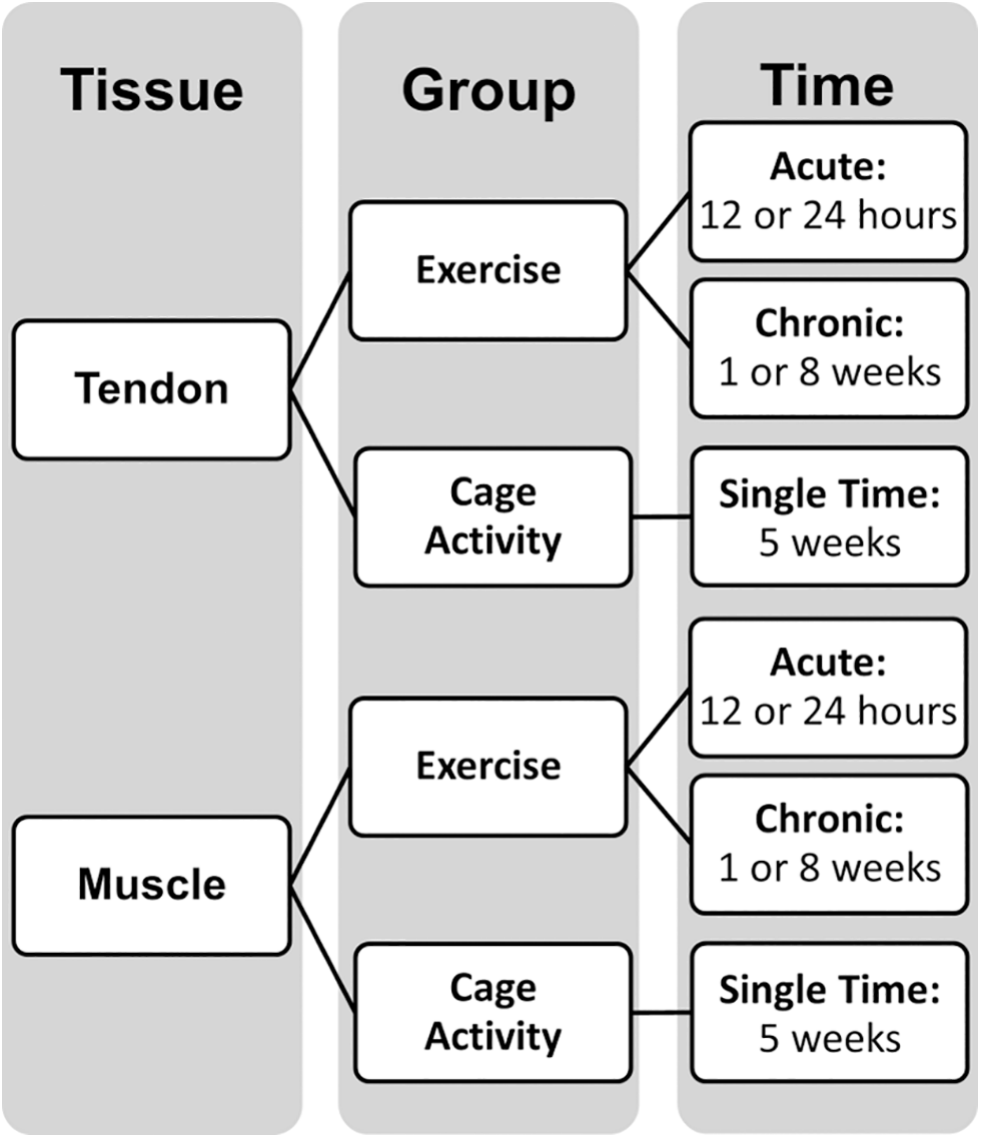
Study design. Supraspinatus tendon and muscle were isolated from rats that had undergone a single bout (12 or 24 hours after) or repeated bouts (1 or 8 weeks) of exercise. Control cage activity rats were sacrificed at a single time point to be within 10% body weight of other groups.

Twenty male, Sprague-Dawley rats (400-450g) were distributed evenly between cage activity (CA) and acute or chronic exercise (EX) groups. The rat shoulder has previously been shown to mimic many of the key features of the human shoulder [6], making it a suitable animal model for this study. To account for potential body weight loading effects on musculoskeletal tissues, animals were ordered within a small weight range (rather than age) that corresponds to ~15 weeks of age [7]. Acute groups were further divided into a 12 or 24 hour euthanasia time point following a single bout of loading, and chronic groups were further divided into 1 or 8 weeks of repeated loading (n = 4 each, Fig 1). Control CA animals maintained normal cage activity for 5 weeks total to reach a final weight within 10% of the groups investigated. Acute EX animals underwent 2 weeks of progressive downhill treadmill training to acclimate to the treadmill. After 72 hours of rest, animals underwent a single treadmill exercise session at a constant speed of 10 meters/minute for 1 hour on a flat treadmill. At 10 meters/minute, rats are walking at only 10–15% of their galloping speed [8,9]. Acute EX animals were euthanized 12 or 24 hours after completion of their single bout of exercise.

After 2 weeks of flat treadmill acclimation, chronic EX animals walked on a flat treadmill at a constant speed of 10 meters/minute for 1 hour per day during normal work hours, 5 days per week, for 1 or 8 weeks. This protocol has previously been shown to produce beneficial, gross adaptations in the rat supraspinatus without inducing tendon mechanical injury [10]. Rats were euthanized 72 hours after their final treadmill session to avoid potentially confounding acute effects of exercise. The treadmill (Nordic Track C2155, Logan, UT, USA) was custom-geared to allow for slower speeds. The treadmill was kept flat, and the speed was checked with a digital handheld tachometer (HT-346, Ono Sokki, Japan) during every exercise session.

At sacrifice, supraspinatus muscle and tendon was harvested from one shoulder of each rat and flash frozen in liquid nitrogen. Tissue specimens were stored at -80°C until RNA extraction.

### RNA Extraction

Tendon and muscle total RNA was extracted using commercially available kits and the TRIzol isolation system for fibrous tissue. Briefly, tissue was manually homogenized via disruption with mortar and pestle. 1 ml of TRIzol Reagent (Life Technologies, Carlsbad, CA, USA) was added for cell lysis and dissociation of nucleoprotein complexes. Homogenized samples were centrifuged for 10 minutes at 4°C at 12,000xg. Supernatant was transferred to a new tube, and 100μl 1-bromo-3-chloropropane was added for phase separation. The aqueous phase where the RNA resides was transferred to a new tube. Molecular grade ethanol was added to the sample. Tendon RNA solutions were cleaned using a Zymo Clean & Concentrator-5 Kit (Zymo, Irvine, CA, USA) with addition of DNAse I (Qiagen, Valencia, CA, USA). Concentrated tendon RNA was eluted using 12 μl DNAse/RNAse-free water. Muscle RNA solutions were loaded into a Zymo DirectZol kit (Zymo, Irvine, CA, USA) and the kit protocol with addition of DNAse I (Zymo, Irvine, CA, USA) was followed. Concentrated muscle RNA was eluted using 25μl of DNAse/RNAse-free water. RNA was stored at -80°C.

### RNA Quality and Integrity

RNA quantity and purity was measured with a NanoDrop spectrophotometer (NanoDrop, Wilmington, DE, USA). Dilute RNA solution was placed on the system and 260/280 nm and 260/230 nm UV absorbance was measured. RNA integrity was assessed with an Agilent Bioanalyzer (Agilent Technologies, Santa Clara, CA, USA) for the purposes of confirming similar RNA quality across specimens from the different groups.

### Panomics QuantiGene 2.0 Multiplex Assay

The purified RNA samples were run on a custom Panomics QuantiGene 2.0 Multiplex array (Affymetrix, Santa Clara, CA, USA) using Luminex xMAP technology (Luminex Corp., Austin, TX, USA). This plate was designed by the manufacturer with probes to simultaneously detect 48 target genes representative of inflammation (arachidonic acid pathway and inflammatory cytokines), extracellular matrix components, matrix turnover enzymes, tissue-specific markers, and factors associated with tendon degeneration and 3 housekeeping genes in each well (Table 1). The 3 housekeeping genes (*Polr2a*, *Ppib*, and *Rplp0*) were chosen based on literature and were expected to be invariant among these groups in these tissues. Housekeeping genes known from the literature to be regulated in muscle and tendon due to exercise (e.g., *Gapdh*, *Pgk1*, *Ldha*) were avoided. These selected three housekeeping genes were also chosen, per manufacturer’s suggestion, to represent a range of expression levels (medium, medium high, and high) to avoid saturating the array signal. After testing dilutions of a tendon and muscle RNA sample, it was determined that 500 ng of muscle RNA and 300 ng of tendon RNA allowed for good signal detection while maintaining signal linearity without saturation. Three wells were used as water blanks for background signal detection. The assay was run following the manufacturer’s protocol for purified RNA.

**Table 1:** Target Genes and Accession Numbers.

Broad Category	Gene	Symbol	Accession
**Inflammation: Arachidonic Acid Pathway**	COX-1	*Ptgs1*	NM_017043
	COX-2	*Ptgs2*	NM_017232
	Arachidonate 5-lipoxygenase activating protein (FLAP)	*Alox5ap*	NM_017260
	PGE synthase	*Ptges*	NM_021583
	PGE receptor 4	*Ptger4*	NM_032076
	PGF receptor	*Ptgfr*	NM_013115
**Inflammation: Cytokines**	Interleukin 1α	*Il1a*	NM_017019
	Interleukin 1β	*Il1b*	NM_031512
	Interleukin 6	*Il6*	NM_012589
	Interleukin 10	*Il10*	NM_012854
	Chemokine (C-X-C motif) ligand 1	*Cxcl1*	NM_030845
	Tumor necrosis factor	*Tnf*	NM_012675
	Nuclear factor -κB	*Nfkb1*	NM_001276711
	Tachykinin (substance P)	*Tac1*	NM_012666
**Matrix: MMPs and TIMPs (turnover)**	MMP2	*Mmp2*	NM_031054
	MMP3	*Mmp3*	NM_133523
	MMP9	*Mmp9*	NM_031055
	MMP13	*Mmp13*	NM_133530
	MMP14	*Mmp14*	NM_031056
	TIMP1	*Timp1*	NM_053819
	TIMP2	*Timp2*	NM_021989
	TIMP3	*Timp3*	NM_012886
	TIMP4	*Timp4*	NM_001109393
**Matrix: Collagen**	Collagen, type I	*Col1a1*	NM_053304
	Collagen, type II	*Col2a1*	NM_012929
	Collagen, type III	*Col3a1*	NM_032085
**Matrix: Proteoglycans**	Decorin	*Dcn*	NM_024129
	Biglycan	*Bgn*	NM_017087
	Aggrecan	*Acan*	NM_022190
	Fibromodulin	*Fmod*	NM_080698
	Versican	*Vcan*	NM_053663
**Growth Factors**	Transforming growth factor β1	*Tgfb1*	NM_021578
	Transforming growth factor β3	*Tgfb3*	NM_013174
	Connective tissue growth factor	*Ctgf*	NM_022266
	Insulin-like growth factor 1	*Igf1*	NM_178866
**Tissue Remodeling and Adhesion Glycoproteins**	Fibronectin 1	*Fn1*	NM_019143
	Tenascin C	*Tnc*	NM_053861
**Tissue-Specific: Cartilage**	SRY-box9 (SOX9)	*Sox9*	XM_003750950
**Tissue-Specific: Bone**	Bone morphogenetic protein 2	*Bmp2*	NM_017178
	Bone morphogenetic protein 7	*Bmp7*	NM_001191856
**Tissue-Specific: Muscle Adaptation**	Peroxisome proliferator-activated receptor gamma, coactivator 1α (PGC-1α)	*Ppargc1a*	NM_031347
**Tendon Degeneration: Apoptosis**	Caspase 3	*Casp3*	NM_012922
	Caspase 8	*Casp8*	NM_022277
**Tendon Degeneration: Heat Shock Proteins**	Heat shock protein 27	*Hspb1*	M86389
	Heat shock protein 70	*Hspa2*	NM_021863
**Tendon Degeneration: Vascularization**	Vascular endothelial growth factor A	*Vegfa*	NM_031836
**Tendon Degeneration: NO Synthase**	Nitric oxide synthase 2, inducible	*Nos2*	NM_012611
	Nitric oxide synthase 3, endothelial	*Nos3*	NM_021838
**Housekeeping**	Peptidylprolyl isomerase B (cyclophilin B)	*Ppib*	NM_022536
	RNA Polymerase II (DNA directed) polypeptide A	*Polr2a*	XM_343922
	Ribosomal protein, large, P0	*Rplp0*	NM_022402

### Statistical Analysis

Study design, data analysis, and interpretation were performed in collaboration with an experienced microarray bioinformatics specialist (JWT). Average background signal of the three water blanks was subtracted from the raw signal intensity values for each gene of each sample. Standard deviation of the background was calculated for each gene. Per the manufacturer’s recommendation, any target with signal less than 3 standard deviations above background (water mean) did not meet the limit of detection and was removed from the analysis. Target gene signal was then normalized by the geometric mean of the three housekeeping genes (*Rplp0*, *Ppib*, *Polr2a*) for each sample. Using the geometric mean of 3 housekeeping genes smooths any bias from a single housekeeping gene and provides a more robust normalization method. The normalized results were log_2_ transformed.

Principal components analysis (PCA) was performed using Partek Genomics Suite (v6.6, Partek Inc., St. Louis, MO) on the entire data set to visualize global similarities among the 40 samples. Additionally, PCA was performed on the four separate categories of interest to visualize separation based on subsets of samples within particular groups: chronic tendon, chronic muscle, acute tendon, and acute muscle. The same cage activity specimens were used for chronic and acute groups. Biologic conclusions are not drawn from these visual representations of the data, but rather these figures are provided to the reader as support for the study design.

This experiment was designed as a screening study to provide important data for the field and to develop avenues for further study. We selected a sample size of n = 4 per group for the benefit of including multiple time points for investigation. Without knowing the expected variation across all 48 target genes and experimental groups in advance, a precise, meaningful power analysis could not be performed. We recognize that for some genes/contrasts this study is underpowered; however, for other genes/contrasts this study is appropriately powered.

For each of the four categories, a 1-way ANOVA was performed using the normalized, log_2_ transformed data to statistically compare CA and EX genes separately for acute and chronic time points and muscle and tendon tissue. Each ANOVA had a single factor with 3 levels (i.e., CA vs. EX 12 hr vs. EX 24 hr for acute; CA vs. EX 1 wk vs. EX 8 wk for chronic), and Partek was used to calculate the p-values for variation in groups. Pairwise contrasts were performed for each of the 3 pairs of comparisons, yielding p-values and fold changes. Because this experiment was performed for screening purposes, an inclusive analysis was conducted. Significance was set at p ≤ 0.05 and genes with a positive or negative fold change ≥ 1.25 were included. Because only a few genes survived multiple testing correction, which would severely limit the information that could be provided to the field, we chose to broaden the statistical approach. Furthermore, there are instances where small fold changes in transcript levels have significant downstream effects. Since some transcript changes may be more potent than others, we used a low fold change threshold for inclusion of genes and allow the reader to make individual conclusions with the presented data. These liberal criteria allow us to provide the most information to the field and to include additional genes in the discussion for future investigation. Graphs are shown as mean ± standard deviation of the log_2_ transformed data, relative to the cage activity group.

## Results

### RNA Quality and Integrity

Muscle RNA was high purity; 260/280 values ranged from 2.0 to 2.1, and 260/230 values ranged from 1.8 to 2.1 measured with NanoDrop. Tendon RNA showed some impurities; 260/280 values ranged from 1.9 to 2.2, and 260/230 values ranged from 1.1 to 2.2. Bioanalyzer results revealed RNA degradation; however, this degradation was consistent among groups, and the high signal readings from the array provided confidence that meaningful conclusions can be drawn from the results. Since the data are analyzed after normalization to housekeeping genes, as long as signal intensities are high, the results are sound. If RNA was severely degraded, signal intensity would not have been adequate for analysis. Additionally, RNA degradation cannot artificially inflate the statistical analysis, indicating that the results we present and statistical differences we detect do reflect the tissue gene expression changes and are not artifacts from the RNA extraction process.

### Panomics QuantiGene 2.0 Multiplex Assay

Quantitative conclusions about gene expression could not be made for the following targets that were below the limit of detection: *Ptgs2* (tendon and muscle), *Il1a* (tendon and muscle), *Il1b* (tendon and muscle), *Il6* (tendon and muscle), *Il10* (tendon and muscle), *Tac1* (tendon and muscle), *Mmp3* (muscle), *Mmp9* (tendon and muscle), *Mmp13* (tendon and muscle), *Col2a1* (muscle), and *Acan* (muscle).

#### Principal Components Analysis

**Fig 2:**
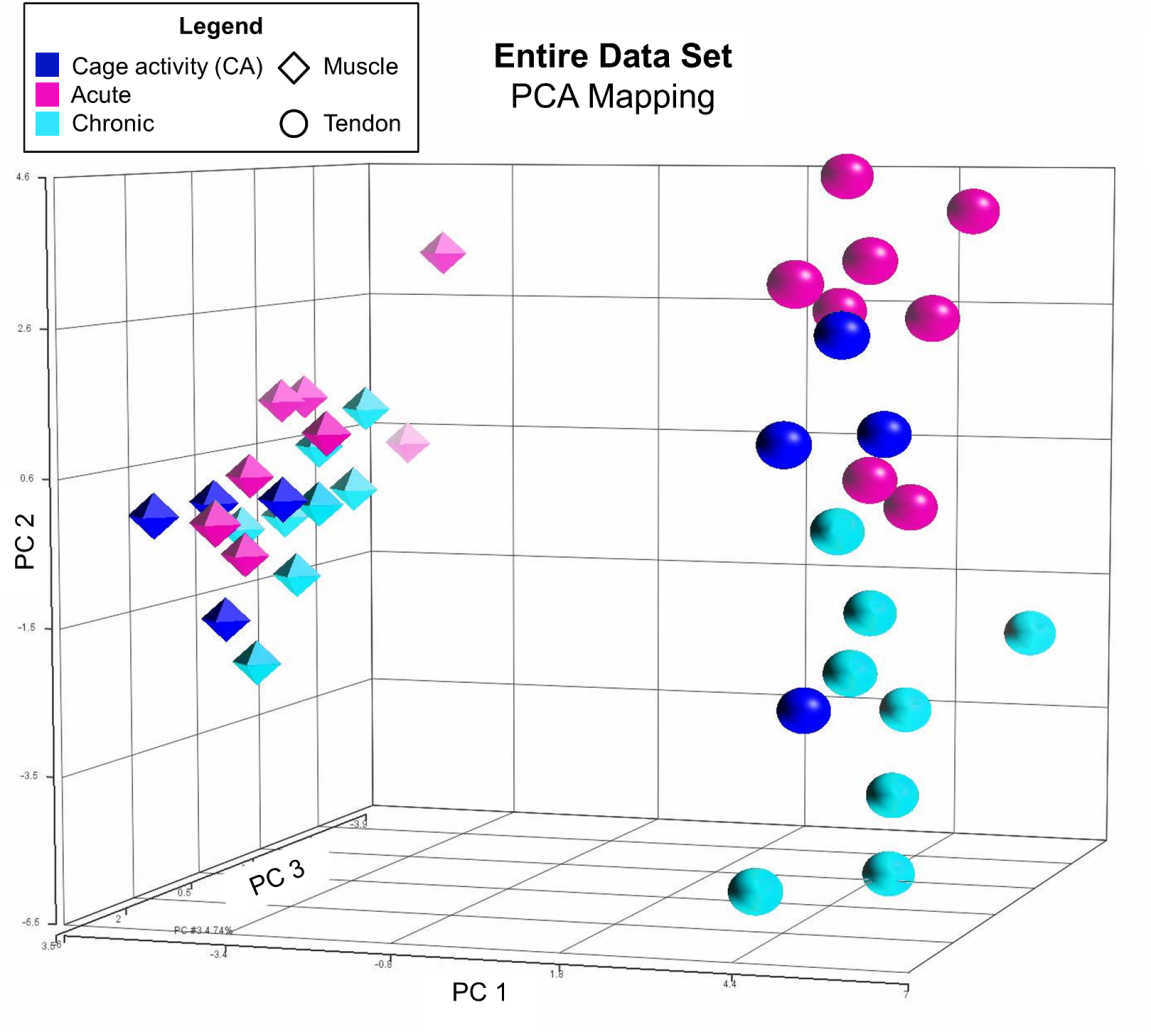
PCA of Entire Data Set. Principal components analysis of the entire data set revealed clear distinctions between muscle/tendon and acute/chronic groups, confirming that muscle and tendon tissues were isolated from each other, and 72 hours of rest prior to euthanasia in the chronic groups provided enough time to avoid potentially confounding acute effects of exercise. The total variation explained by PCs 1, 2, and 3 is 71.9%.

**Fig 3:**
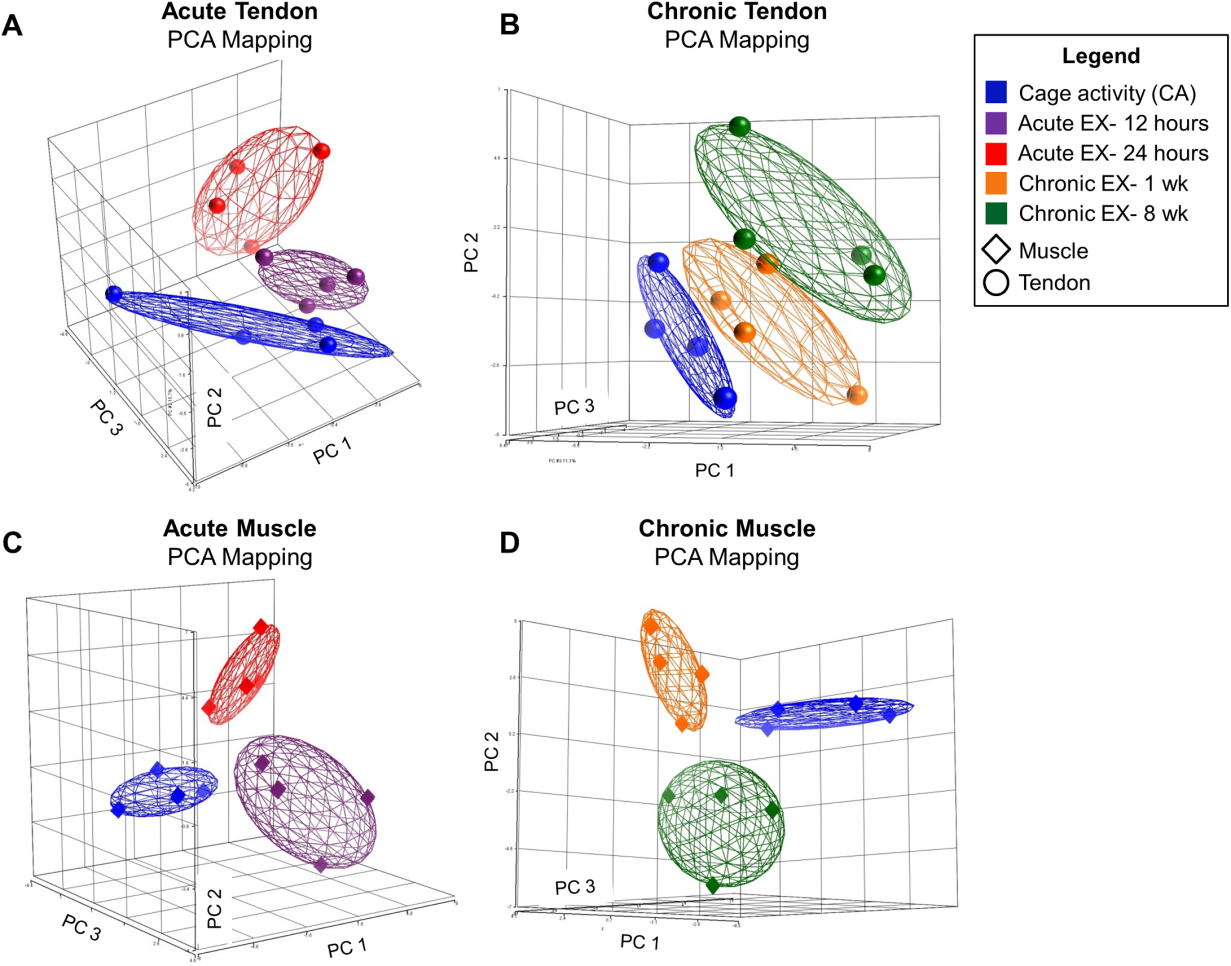
PCA of Each Category of Interest. Principal components analysis of the 4 categories of interest show distinct clusters that correspond to each of the 3 groups (levels) within the category, supporting the study design. Figs are of individual data points with 1.5 standard deviation ellipsoids. A) Acute Tendon. The total variation explained by PCs 1, 2, and 3 is 58.8%. B) Chronic Tendon. The total variation explained by PCs 1, 2, and 3 is 60%. C) Acute Muscle. The total variation explained by PCs 1, 2, and 3 is 63.9%. D) Chronic Muscle. The total variation explained by PCs 1, 2, and 3 is 60.5%.

PCA of the entire sample pool revealed the greatest variance between muscle and tendon specimens, confirming that these two tissues were isolated from each other during dissection (Fig 2). Additionally, a clear distinction could be seen between acute and chronic groups, suggesting that acute and chronic processes differ and that 72 hours of rest prior to tissue harvest in the chronic groups provided enough time to avoid potentially confounding acute effects of exercise. Finally, and importantly, individual PCA of each of the four categories revealed distinct clusters corresponding to each of the 3 groups (levels) within the category (Fig 3).

#### Acute Tendon

**Fig 4:**
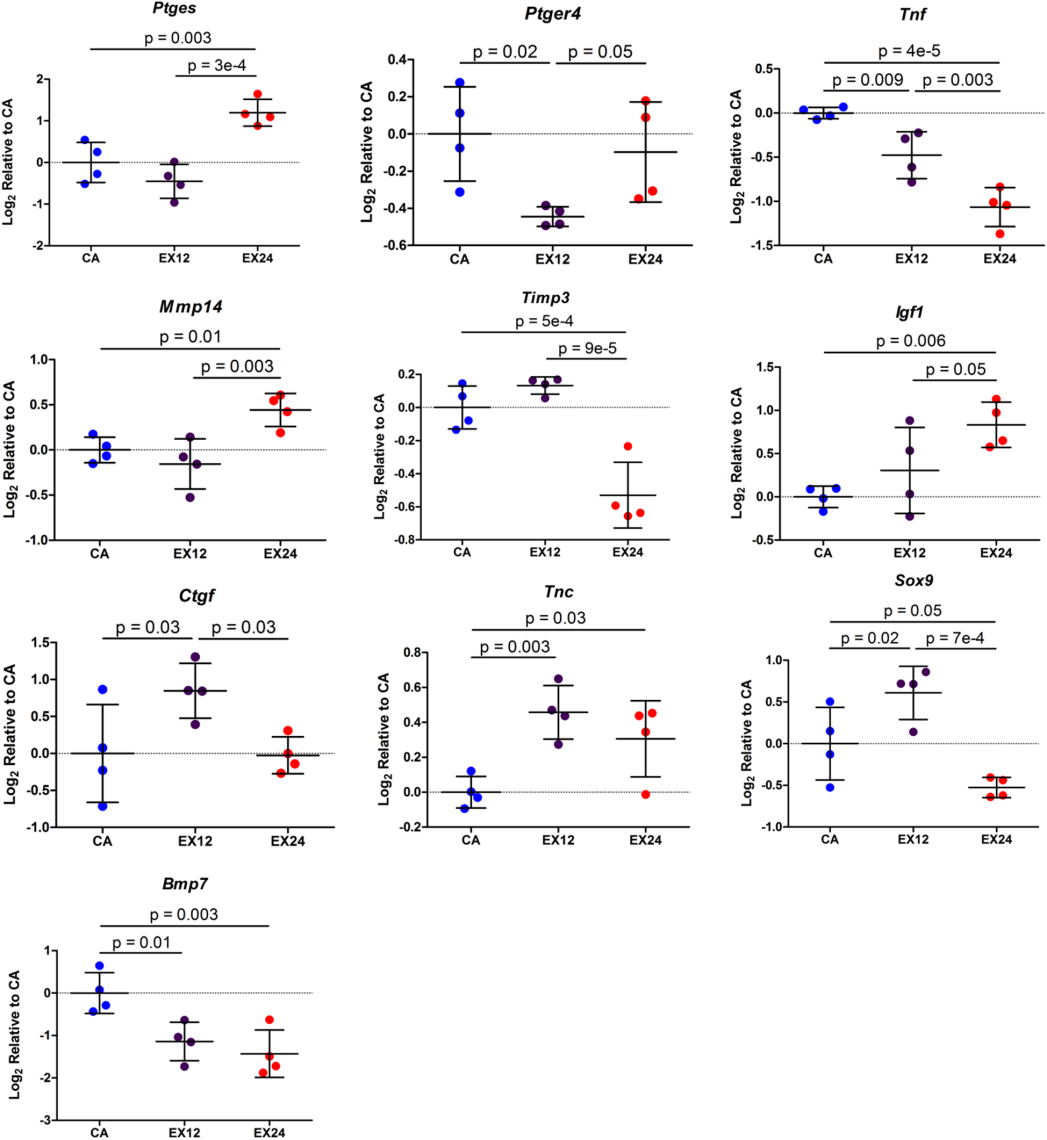
Significant Target Gene Changes in Tendon with Acute Exercise. Changes in gene expression of inflammatory-related factors *Ptges*, *Ptger4*, and *Tnf* and matrix enzymes *Mmp14* and *Timp3* suggest that a single bout of non-injurious exercise regulates inflammatory processes and matrix turnover in supraspinatus tendon. Mean ± Standard Deviation.

For the acute tendon category, 10 target genes were identified as having an ANOVA p-value ≤ 0.05: *Ptges*, *Ptger4*, *Tnf*, *Mmp14*, *Timp3*, *Igf1*, *Ctgf*, *Tnc*, *Sox9*, and *Bmp7* (Fig 4). Pairwise comparisons indicated downregulation of *Ptger4*, *Tnf*, and *Bmp7* and upregulation of *Ctgf*, *Tnc* and *Sox9* 12 hours after an acute bout of exercise. 24 hours following an acute bout of exercise, pairwise comparisons revealed downregulation of *Tnf*, *Timp3*, *Sox9*, and *Bmp7* and upregulation of *Ptges*, *Mmp14*, *Igf1*, and *Tnc*. *Tnc* expression 24 hours following an acute bout of exercise compared to cage activity did not meet the 1.25 fold change criteria but is presented on the graph with p-value for completeness; all other significant pairwise contrasts had a positive or negative fold change ≥ 1.25.

#### Acute Muscle

**Fig 5:**
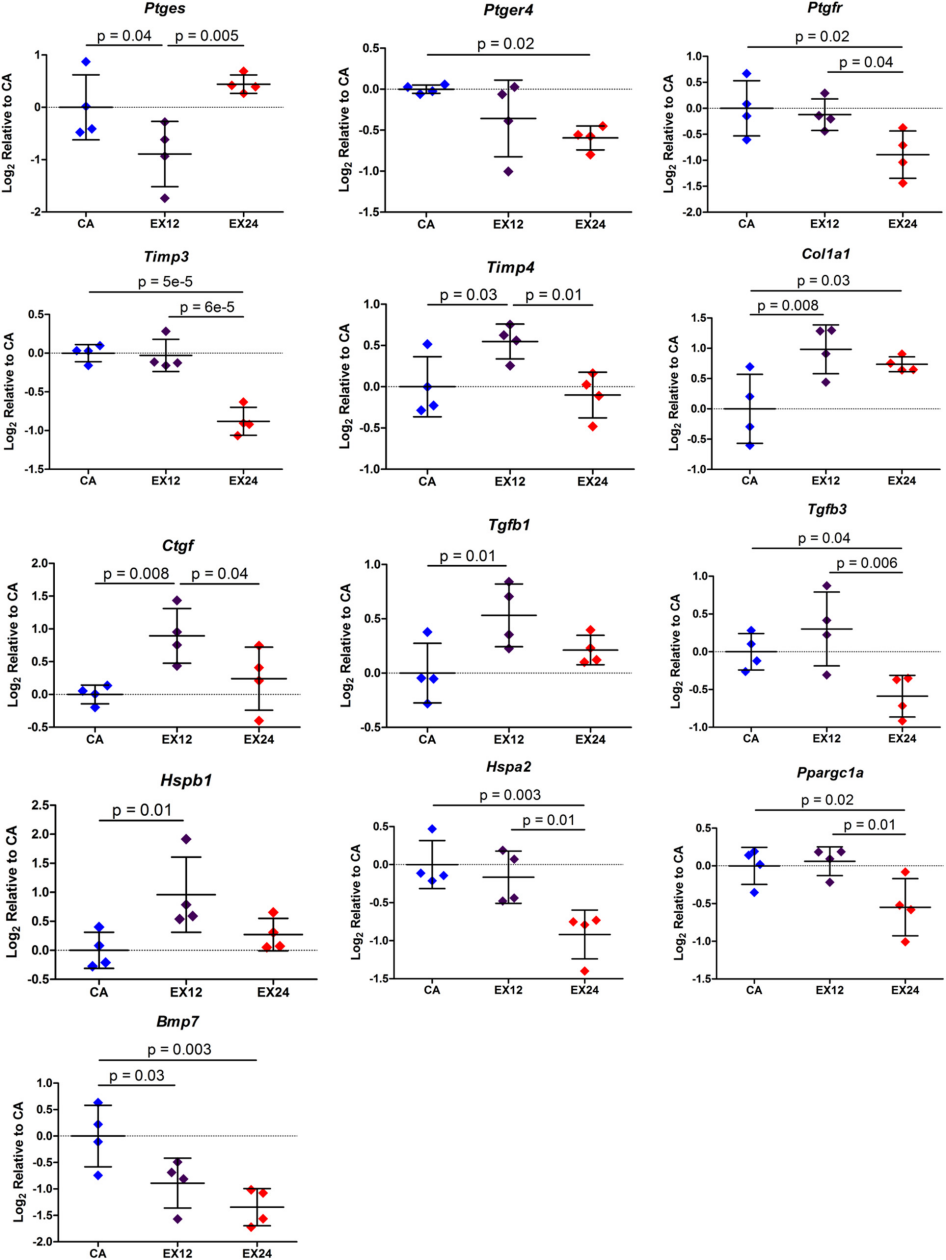
Significant Target Gene Changes in Muscle with Acute Exercise. Changes in gene expression of arachidonic acid cascade components *Ptges*, *Ptger4*, and *Ptgfr* and matrix components *Col1a1*, *Timp3*, and *Timp4* suggest that a single bout of non-injurious exercise regulates inflammatory processes and matrix turnover in supraspinatus muscle. Mean ± Standard Deviation.

For the acute muscle category, 13 target genes were identified as having an ANOVA p-value ≤ 0.05: *Ptges*, *Ptger4*, *Ptgfr*, *Timp3*, *Timp4*, *Col1a1*, *Ctgf*, *Tgfb1*, *Tgfb3*, *Hspb1*, *Hspa2*, *Ppargc1a*, and *Bmp7* (Fig 5). Pairwise comparisons indicated downregulation of *Ptges* and *Bmp7* and upregulation of *Timp4*, *Col1a1*, *Ctgf*, *Tgfb1*, and *Hspb1* 12 hours after an acute bout of exercise. 24 hours following an acute bout of exercise, pairwise comparisons revealed downregulation of *Ptger4*, *Ptgfr*, *Timp3*, *Tgfb3*, *Hspa2*, *Ppargc1a*, and *Bmp7* and upregulation of *Col1a1*. All significant pairwise contrasts had a positive or negative fold change ≥ 1.25.

#### Chronic Tendon

**Fig 6:**
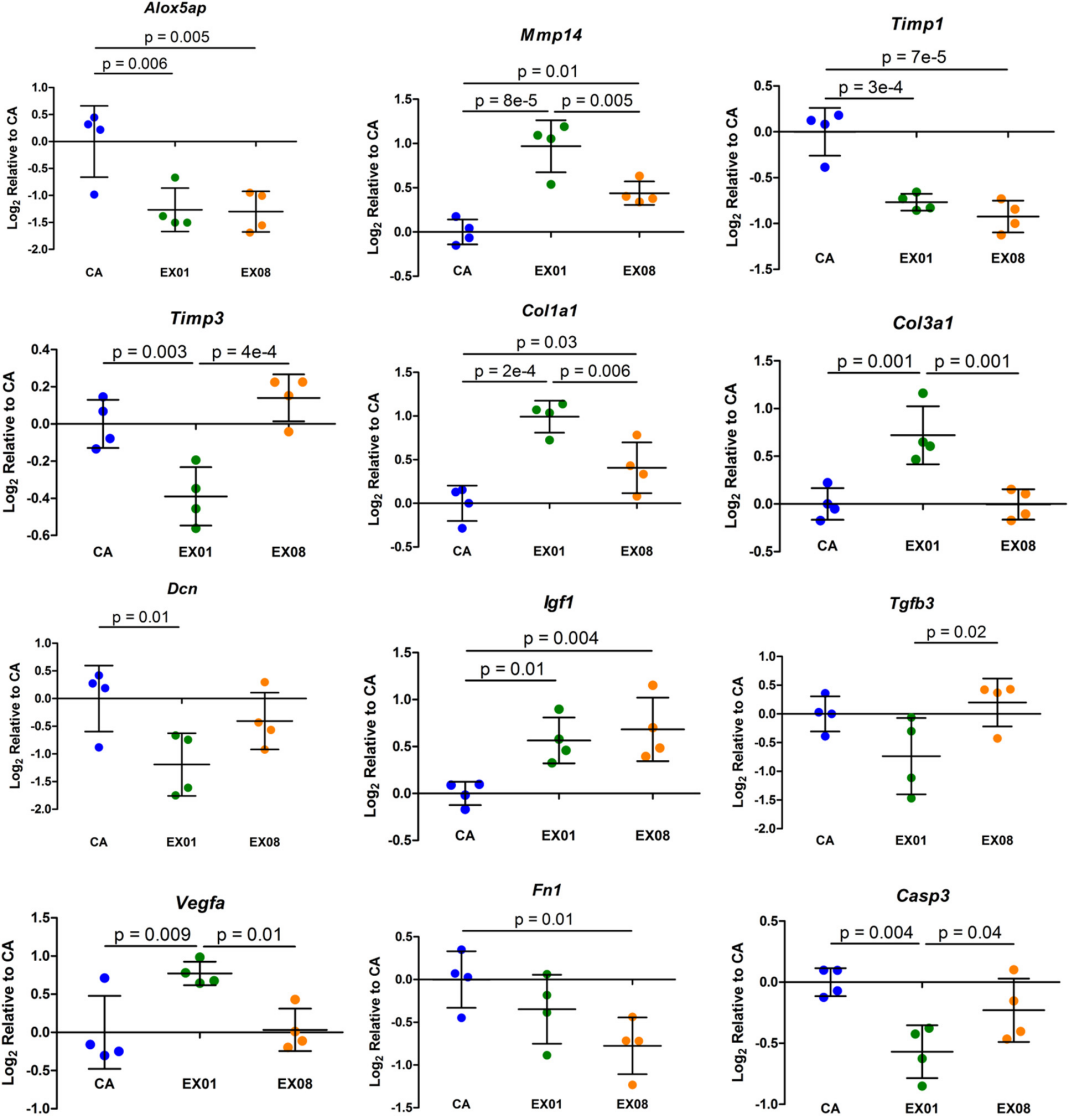
Significant Target Gene Changes in Tendon with Chronic Exercise. Changes in collagen type I and III expression, decorin, and matrix turnover enzymes *Mmp14*, *Timp1*, and *Timp3* indicate an adaptive matrix response in the supraspinatus tendon to non-injurious, chronic exercise. Compared to 8 weeks of chronic exercise, more gene changes are seen at 1 week, suggesting that these adaptive processes begin soon after initiating an exercise protocol. The only altered inflammatory gene (AA pathway or cytokine) was *Alox5ap*, indicating that inflammation associated with the arachidonic acid cascade is not a main pathway in the chronic response of tendon to exercise. Mean ± Standard Deviation.

For the chronic tendon category, 12 target genes were identified as having an ANOVA p-value ≤ 0.05: *Alox5ap*, *Mmp14*, *Timp1*, *Timp3*, *Col1a1*, *Col3a1*, *Dcn*, *Igf1*, *Tgfb3*, *Vegfa*, *Fn1*, and *Casp3* (Fig 6). Pairwise comparisons indicated downregulation of *Alox5ap*, *Timp1*, *Timp3*, *Dcn*, and *Casp3* and upregulation of *Mmp14*, *Col1a1*, *Col3a1*, *Igf1*, and *Vegfa* after 1 week of exercise. After 8 weeks of exercise, pairwise comparisons revealed downregulation of *Alox5ap*, *Timp1*, and *Fn1* and upregulation of *Mmp14*, *Col1a1*, and *Igf1*. Compared to 8 weeks of chronic exercise, more gene changes are seen after 1 week, suggesting that these adaptive processes begin soon after initiating an exercise protocol. All significant pairwise contrasts had a positive or negative fold change ≥ 1.25.

#### Chronic Muscle

**Fig 7:**
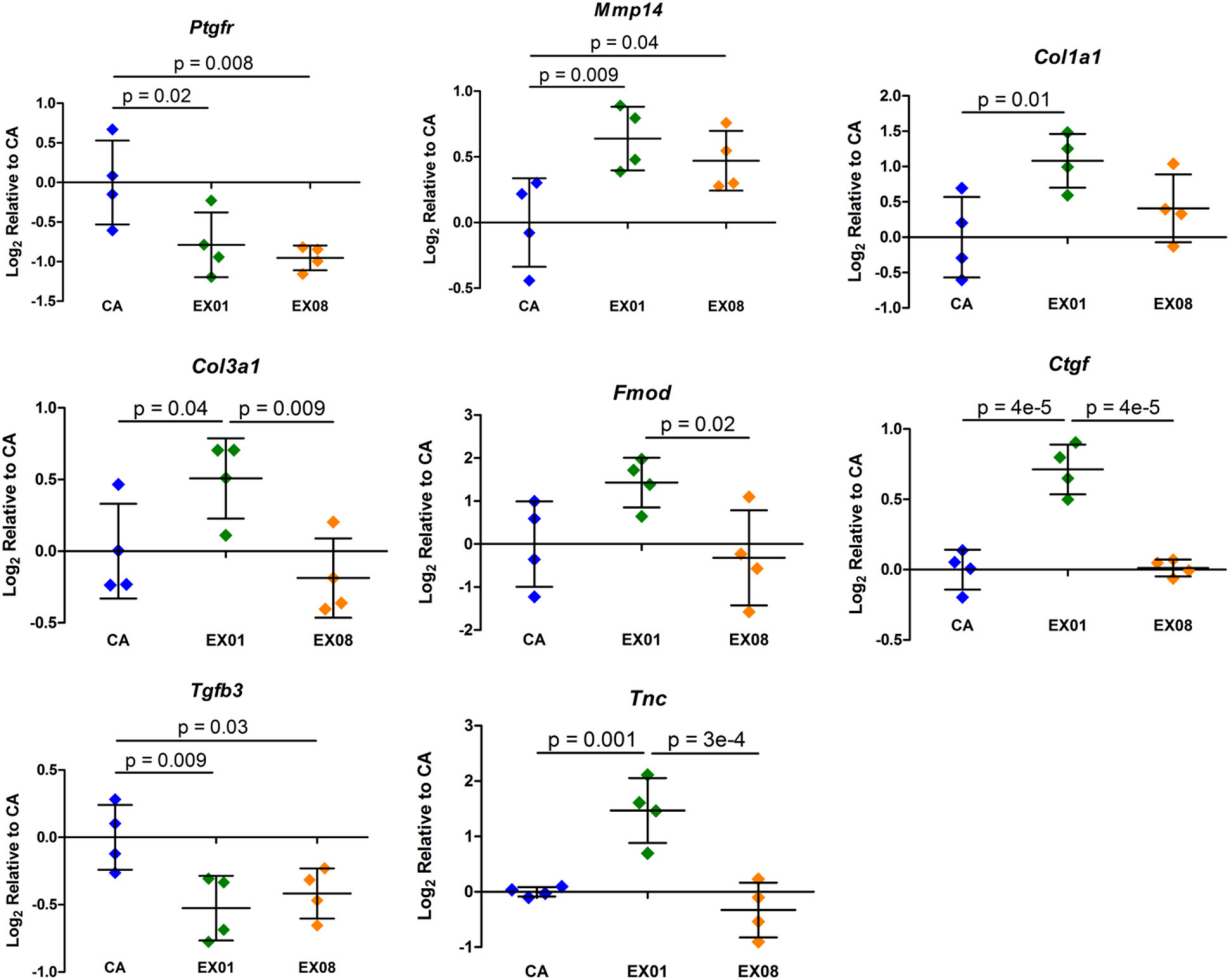
Significant Target Gene Changes in Muscle with Chronic Exercise. Similar to chronic tendon changes, following chronic exercise supraspinatus muscle showed adaptations in the matrix turnover components *Mmp14*, *Col1a1*, and *Col3a1* in addition to *Fmod*. Changes were more pronounced at 1 week than 8 weeks, indicating early adaptation to increased loading. The only altered inflammatory gene (AA pathway or cytokine) was *Ptgfr*, indicating that inflammation is not a main pathway in the chronic response of muscle to exercise. Mean ± Standard Deviation.

For the chronic muscle category, 8 target genes were identified as having an ANOVA p-value ≤ 0.05: *Ptgfr*, *Mmp14*, *Col1a1*, *Col3a1*, *Fmod*, *Ctgf*, *Tgfb3*, and *Tnc* (Fig 7). Pairwise comparisons indicated downregulation of *Ptgfr* and *Tgfb3* and upregulation of *Mmp14*, *Col1a1*, *Col3a1*, *Ctgf*, and *Tnc* after 1 week of exercise. After 8 weeks of exercise, pairwise comparisons revealed downregulation of *Ptgfr* and *Tgfb3* and upregulation of *Mmp14*. Changes were more pronounced after 1 week than 8 weeks of exercise, indicating early adaptation to increased loading. All significant pairwise contrasts had a positive or negative fold change ≥ 1.25.

The altered genes in supraspinatus tendon and muscle following acute and chronic non-injurious exercise are summarized in Table 2, highlighting the numerous inflammatory-related genes that are regulated acutely and numerous matrix-related genes that are regulated chronically.

**Table 2:** Summary of Target Genes Altered with Acute and Chronic Exercise in Tendon and Muscle. Following a single bout of exercise, inflammatory-related genes are altered in muscle and tendon; following chronic exercise, numerous matrix-related genes are altered in muscle and tendon.

	Acute Tendon	Acute Muscle	Chronic Tendon	Chronic Muscle
**Inflammation**	*Ptges*	*Ptges*	*Alox5ap*	*Ptgfr*
	*Ptger4*	*Ptger4*		
	*Tnf*	*Ptgfr*		
**Matrix**	*Mmp14*	*Col1a1*	*Col1a1*	*Col1a1*
	*Timp3*	*Timp3*	*Col3a1*	*Col3a1*
		*Timp4*	*Dcn*	*Fmod*
			*Mmp14*	*Mmp14*
			*Timp1*	
			*Timp3*	
**Growth Factors**	*Igf1*	*Ctgf*	*Igf1*	*Ctgf*
	*Ctgf*	*Tgfb1*	*Tgfb3*	*Tgfb3*
		*Tgfb3*	*Vegfa*	
**Tissue-Specific**	*Bmp7*	*Bmp7*		
	*Sox9*	*Ppargc1a*		
**Tendon Degeneration: Heat Shock Proteins**		*Hspb1*		
		*Hspa2*		
**Tendon Degeneration: Apoptosis**			*Casp3*	
**Remodeling and Adhesion**	*Tnc*		*Fn1*	*Tnc*

## Discussion

This study is novel in that it investigated both the acute and chronic genetic responses of rat supraspinatus tendon and muscle following a non-injurious exercise protocol. PCA confirmed clear distinctions between tissues and between time points supporting the study design and groups for evaluation. Undetectable levels of *Acan* and *Col2a1* in muscle, but not tendon samples, further confirmed isolation of these two tissues during dissection. Tendon and muscle samples showed differential, time-dependent responses to exercise. Similar to other studies, we detected a load-induced alteration of growth factors (*Igf1*, *Ctgf*, *Tgfb1*, *Tgfb3*, *Vegfa*) in muscle and tendon [11]. Chronic, compared to acute, tendon and muscle samples were characterized as having more matrix-related gene changes, suggesting tissue adaptation to chronic loading. Specifically, both supraspinatus muscle and tendon demonstrated increases in collagen type I gene expression and alterations in *Mmp14* and TIMPs, implying that matrix turnover is an important component of tissue adaptation to load. More gene changes were found at the 1 week time point than the 8 week time point, indicating that this adaptive process begins soon upon initiation of an exercise routine. As the tissue adapts over time, it may require a greater loading stimulus to achieve the same gene expression and matrix turnover response. Acutely, genes associated with the arachidonic acid cascade (*Ptges*, *Ptger4*, and *Ptgfr*) were altered in muscle and tendon 12 and 24 hours following a single bout of exercise. Furthermore, changes in *Mmp14* and TIMPs suggest an acute matrix turnover response to exercise in addition to the chronic, adaptive matrix turnover response. Acute and chronic responses of MMPs/TIMPs to exercise have been seen in other studies as well [12,13]. Similar to our study, MMP-14 mRNA in skeletal muscle increased with 10 days of training [12]. Acute changes may play an important role in initiating the downstream processes that lead to beneficial adaptations to exercise. Results of this study suggest that inflammation-related pathways are altered acutely in tendon and muscle following a single bout of exercise; however, the precise timing of this response and when levels return to baseline has not been determined. Furthermore, we detected upregulation, downregulation, and no change in expression of various inflammation and matrix turnover-related genes, so our results cannot conclude whether exercise results in a tissue-level inflammatory response; however, our results do support coordinate, temporal regulation of these pathways in response to exercise, providing support for future investigations on the roles of inflammation and matrix turnover in adaptations to exercise. As expected, inflammatory genes were more changed at acute time points than chronic, and results support the hypothesized adaptive matrix changes following chronic loading.

The Panomics QuantiGene 2.0 multiplex assay used in this study has the benefit of detecting original RNA quantities with high precision and accuracy by using branched DNA signal amplification without reverse transcription, avoiding the bias and false positive and negative results that can be associated with cDNA synthesis and PCR amplification. Furthermore, these arrays have been used successfully on formalin-fixed, paraffin-embedded, hematoxylin and eosin-stained samples that have very poor RNA integrity, providing further support for use of this assay on moderately degraded samples. With this array, genes can be simultaneously detected within a sample. Due to limitations on the size of the plate, only one RNA sample was run per specimen rather than averaging replicates. Furthermore, the sample size was restricted to 4 specimens per group to allow investigation of multiple time points. The differences we found between 12/24 hours and 1/8 weeks support the importance of including multiple time points in this study. A single, untrained cage activity group within 10% of the weight of the other groups was used for all comparisons. Several genes did not meet the limit of detection, despite our best efforts in optimizing the quantity of RNA. Despite these limitations, we detected several gene changes in supraspinatus muscle and tendon suggesting a role for the arachidonic acid cascade and MMPs and TIMPs in the adaptation of tissue to beneficial exercise. This study provides gene transcript level of evidence, and functional changes within a tissue are due to the coordination of transcription, translation, activation, inhibition, and receptors. As with any RNA screening study, future work should seek to confirm protein changes in muscle and tendon associated with acute and chronic responses to exercise.

*In vitro* experiments have shown that cultured human tenocytes respond to strain by modulating production of prostaglandin E2 (PGE_2_), COX-1, COX-2 [14], and leukotriene B(4) [15], implying load-induced activation of the AA cascade in tendon. Despite implications that prostaglandins may contribute to early tendon degeneration [16–18], their presence may indicate tendon remodeling [19], which is supported by increased PGE_2_ levels in humans following a bout of exercise [20,21]. The present study adds further evidence of a physiologic (not pathologic), acute prostaglandin response to beneficial exercise in tendon and muscle. Whether these up and downregulations correspond with increased and decreased protein expression will need to be verified in future studies. Importantly, gene changes occurred as a result of an acute bout of exercise, implying that inflammatory and matrix turnover pathways are regulated in the tissue response to exercise and could lead to activation of downstream processes. PGE_2_ may be involved in converting mechanical load to type I collagen synthesis, the primary component of tendon [1], further supported by the increased tendon and muscle *Col1a1* expression in this study.

The results of this screening study suggest that tendon response to chronic beneficial exercise is distinct from chronic overuse loading. Specifically, following chronic overuse, tendon exhibits a more cartilaginous phenotype [22] with increased type III:I collagen ratio (fibrosis), heat shock proteins [23], and nitric oxide synthases [24]. Following chronic exercise in this study, we did not find increased expression of the cartilage markers *Sox9*, *Acan*, or *Col2a1*, the heat shock proteins *Hspa2* and *Hspb1*, or the nitric oxide synthases *Nos2* and *Nos3* in tendon. After 1 week of exercise, both *Col1a1* and *Col3a1* expression increased; however, by 8 weeks *Col3a1* returned to baseline expression levels, while *Col1a1*, the main structural component of tendon, remained elevated. Increased type I collagen may lead to improved tendon mechanical properties. Finally, chronic overuse tendons demonstrate increased COX-2 and 5-lipoxygenase activating protein gene expression [25]; however, in this non-injurious exercise animal model, 5-lipoxygenase activating protein (*Alox5ap)* gene expression decreased in tendon with chronic exercise, and COX-2 (*Ptgs2)* was below the limit of detection. These results indicate differential responses of the inflammatory cascade in tendon following chronic overuse compared to non-injurious exercise.

In conclusion, this study suggests that inflammation-related processes and matrix turnover in supraspinatus muscle and tendon are regulated by non-injurious exercise both acutely and chronically, providing important data for the community that can be used for further investigation. Future studies can use these results to distinguish beneficial and detrimental loading effects on tissue, identify tissue recovery, and develop new treatment options.
